# Technical considerations for medical device manufacturers when designing gastrostomy tubes (G-tubes) using the new ISO 80369-3 connector

**DOI:** 10.1371/journal.pone.0236644

**Published:** 2020-07-30

**Authors:** Suvajyoti Guha, Alexander Herman, Luke Herbertson, Mark J. Antonino, Joshua S. Silverstein, Jeffrey Cooper, Matthew R. Myers

**Affiliations:** 1 Office of Science and Engineering Laboratories, Center for Devices and Radiological Health, U.S. Food and Drug Administration, Silver Spring, Maryland, United States of America; 2 Office of Product Evaluation and Quality, Center for Devices and Radiological Health, U.S. Food and Drug Administration, Silver Spring, Maryland, United States of America; University of Glasgow, UNITED KINGDOM

## Abstract

**Background:**

Gastrostomy tubes (G-tubes) are typically used when people cannot eat food by mouth. The connector section that allows G-tubes to connect to other devices, such as feeding sets or syringes, has been modified on some of the devices to reduce misconnections in hospital settings. The narrow internal diameter of the new connector, standardized under ISO 80369–3, has caused some users to express concern about a reduced flow rate. Previous studies performed on commercial devices determined that it was not conclusive how much the ISO 80369–3 connector contributed towards the reduced flow rate, because when manufacturers designed these new connector-based devices, they often changed other geometric variables (such as distal tube diameter, or length) at the same time. Thus, it became difficult isolating the effect of the connector from other geometric variables.

**Method:**

The key objective of this study was to investigate how different design variables impacted the flow rate through the G-tubes. 3D-printed devices were used to assess the geometric parameters in a systematic manner. Commercial diets and Newtonian analog fluids with matched viscosities were used for testing.

**Results:**

The flow path length of the “transition section” encompassing the standardized ISO 80369–3 connector in the new devices was found to cause reduced flow. Additionally, results showed that a shortened (≤ 10 mm) transition section, along with a 10% increase in the distal inner diameter of large bore devices (e.g., 24 Fr), can restore flow rates to levels consistent with the previous devices prior to the connector standardization.

**Conclusions:**

The strategy for restoring flow rates to previous levels may help alleviate concerns raised by multiple stakeholders such as health care professionals, patients, caregivers and device manufacturers. In addition, the approach proposed here can be used as a tool for designing future G-tube devices.

## Introduction

**Fig 1:**
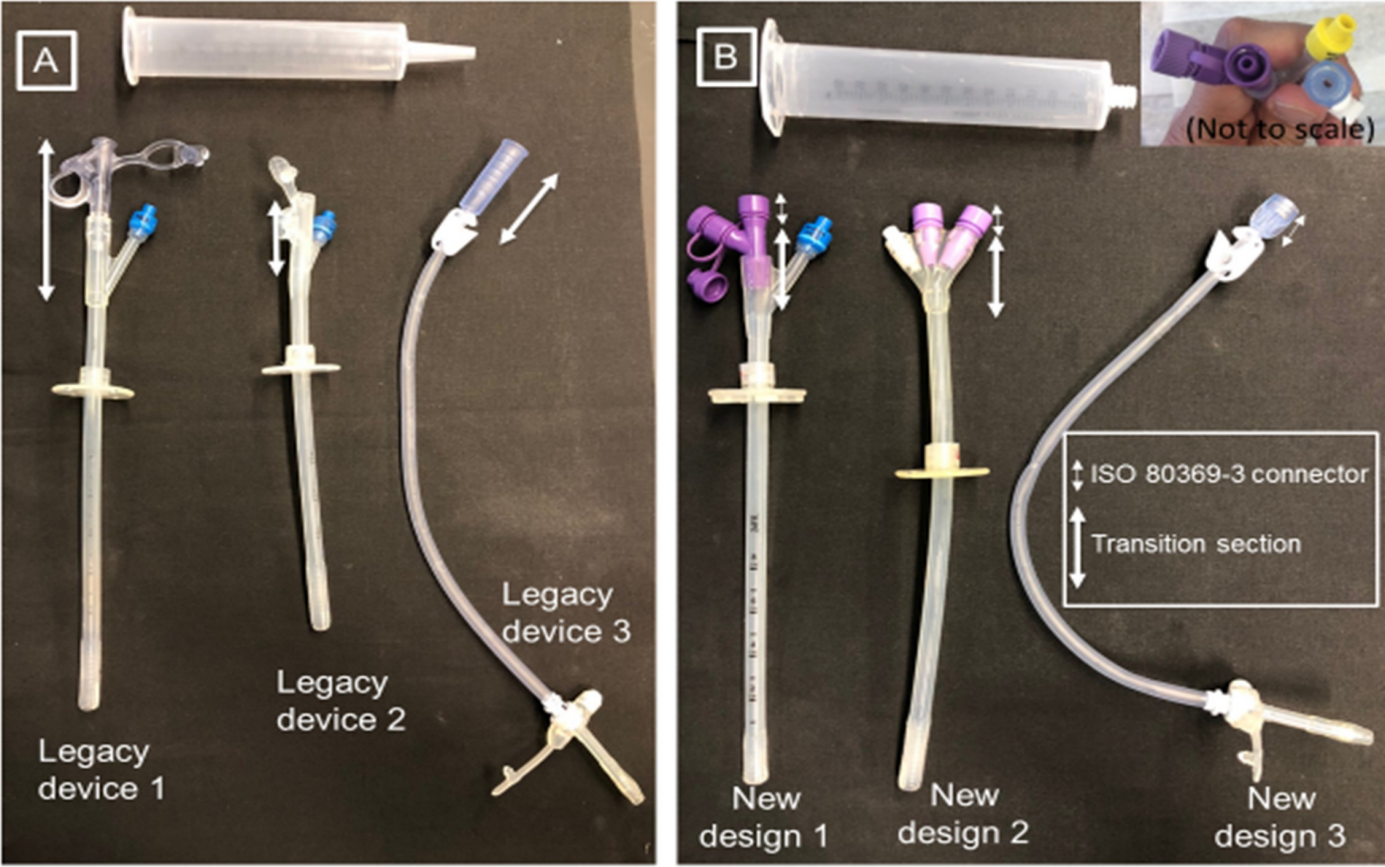
Existing devices and different transition scenarios. (A): A legacy syringe is shown on top with the plunger of the syringe removed. At the bottom are three different legacy G-tubes currently in the U.S. market. All G-tubes have funnel type connectors. (B) An ISO 80369–3 compliant syringe is shown on top with the plunger of the syringe removed. At the bottom are three newer G-tubes with ISO 80369–3 compliant connectors. The inset shows a top view of the ISO 80369–3 compliant connector for two different designs. The numbering of devices in Fig 1 are for explanation purposes only and do not necessarily reflect the devices from our prior study [6].

Enteral feeding tubes that are inserted percutaneously through the abdominal wall into the stomach, or G-tubes, are Class II medical devices [1]. These devices are used for delivering nutrition to people who otherwise would have poor voluntary intake by mouth, chronic neurological or mechanical dysphagia, metabolic disorders, or are critically ill [2]. Annually, there are 500,000 patients in the United States needing G-tubes [3]. Many of these medical devices have recently been modified to minimize the risks of misconnections in hospital settings by using small-bore connectors that cannot connect with other healthcare applications [4]. Fig 1A shows a few commercially available devices. They have two common features: (1) a funnel type transition section that interfaces with a syringe or other medical equipment, and (2) a distal section. The connector section can be wide (approximately ≥5 mm, as in designs 1 and 3 in Fig 1A) or narrow (~3 mm as in design 2 in Fig 1A). The ability of the funnel-type connector to accommodate different-sized male connections has resulted in adverse events in hospital settings attributed to misconnections. To reduce misconnections, several G-tube manufacturers have redesigned their devices to include a small bore connector that standardizes the connector internal diameter to 2.9 mm (ISO 80369–3). Fig 1B shows three such modified devices. These new G-tube devices have a short ISO 80369–3 connector, and possibly a transition section. The syringes associated with the G-tubes are also shown at the top of Fig 1A and 1B.

Some patients raised concerns that the new ISO 80369–3 design with its 2.9 mm internal diameter (Fig 1B) is smaller than the internal diameter in the connection section of some legacy designs (e.g. the first device shown in Fig 1A). G-tube users were concerned that their quality of life might be impacted by a design change. For instance, slower gravity-driven flows would increase feeding times.

To understand the impact of the ISO 80369–3 connector on the performance of G-tubes, FDA and Mayo Clinic independently performed laboratory tests [5,6] with the legacy and newer devices designed with the ISO 80369–3 connector. In about 70% of the tests, the newer ISO 80369–3 devices were found to be statistically slower than legacy devices [6]. However, when manufacturers redesigned their G-tubes they often changed multiple geometric variables, including the total transition length, the internal diameter in the distal section, and the overall length of the device, all of which can impact the flow rate of blended diets. Due to multiple design changes implemented simultaneously, it was difficult to isolate the impact of individual device modifications such as the ISO 80369–3 connector on the flow through the newer G-tubes. In this article, we used surrogate devices with controlled geometries to investigate the extent to which different design variables can reduce the flow rate, with the objective of providing specific technical information that could be assessed by medical device manufacturers. Given that there is currently no publicly available G-tube design methodology, the methodology proposed here can also be adopted for designing future G-tube designs.

## Materials and methods

### Surrogate devices and their designs

**Fig 2:**
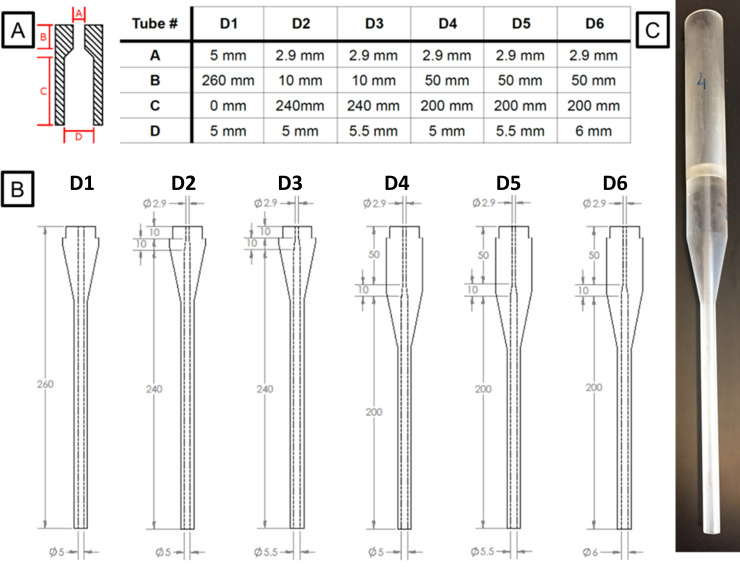
Computer aided and 3D printed designs. (A) Shows the critical design parameters of the designs chosen. A: inlet tube diameter, B: length of the connector, C: length of the surrogate G-tube devices from where the connector ends to the distal end, D: outlet tube diameter. (B) SolidWorks designs of the six surrogate designs showing the internal passageways. All dimensions are in mm. (C) 3D printed D4 syringe-design combination is shown (not to scale).

We designed and fabricated surrogate devices with dimensions that would approximately match the 24 Fr legacy and ISO 80369–3 devices. Devices were fabricated to isolate the effects of transition section including the connector length and distal internal diameter. Six surrogate devices (D1-D6), with major dimensions identified (Fig 2A), and displayed in Fig 2B, were designed in SolidWorks (Dassault Systèmes, Waltham, MA) and then 3D-printed (Connex3 Objet260, Stratasys Ltd., Eden Prairie, MN) using RGD835 (Stratasys Ltd., Eden Prairie, MN) for the base material and SUP706 (Stratasys Ltd., Eden Prairie, MN) for the support material. D1, a baseline design, corresponds to legacy design 1. D2 and D3 are perturbations of D1, accommodating the smaller connector diameter of standard ISO 80369–3 with D3 having a slightly increased distal internal diameter (5.5 mm vs. 5 mm). D4 contains a longer inlet section (50 mm instead of 10 mm) and is like the legacy design 2 in Fig 1A and newer ISO 80369–3 designs 1 and 2 in Fig 1B. Surrogate designs D5 and D6, constructed to investigate the advantages of increasing the distal diameter, are similar to D4 but with the distal section slightly modified (5.5 mm and 6 mm, respectively, compared to 5 mm for D4). To eliminate regions of flow separation observed in some prototype designs, a 10 mm gradual taper was introduced in surrogates D2 to D6. For each of the six surrogate device designs, a top reservoir was also implemented to mimic flow through a syringe. Fig 2A and 2B shows the various dimensions for all the geometries. Fig 2C shows the entire 3D-printed unit along with the syringe surrogate for D4.

### Fluids used for testing

Three liquids, water, Osmolite™ 1.0 (Abbott Nutrition, Columbus, OH) and Boost™ (Very High Calorie) (Nestlé HealthCare Nutrition Inc., Florham Park, NJ) were used to determine flow rate ranges through the surrogate devices. In our previous study [6], we found that Osmolite™ and Boost™ are both shear thinning (viscosity decreases with amount of shearing in the fluid) and have a viscosity of approximately 10 cP and 100 cP at a clinically relevant shear rate of 100 s^-1^. To help identify any shear thinning effects during flow, three Newtonian surrogate fluids were made with viscosities of approximately 10, 100 and 200 cP at room temperature (22.5^o^ +/- 1°C). These test fluids were comprised of specific formulations of glycerin (99.7% USP Kosher, ChemWorld, Atlanta, GA) and deionized, ultrapure water. The aqueous glycerin solutions were well-mixed for at least 5 minutes prior to testing and made in batches of up to 2.3 L at a time. The 10 cP test fluid, referred to as 10 cP g-w (glycerin-water), approximately matches Osmolite™’s viscosity at the clinically relevant shear rate of 100 s^-1^, while the 100 cP viscosity test fluid referred to as 100 cp g-w represents that of Boost™ at the same shear rate.

### Experiments

The surrogate G-tube-syringe combination was filled to a specific volume of approximately 60 mL by pouring the diets from above. This filling method mimics gravity type feeding that is commonly practiced by many patients [6]. The distal end of the device was blocked manually prior to testing to prevent flow. A timer (Stock Stopwatch application, Apple iOS, Cupertino, CA 95014) with a precision of 0.001 seconds was manually started simultaneously with the flow through the device. Once the entire volume was dispensed, the timer was manually stopped and the dispensing time was recorded in seconds (s). The experiments were performed at room temperature (22.5^o^ ± 1°C). All experiments were conducted in triplicate for the different diets with different fluid viscosities. The Student t-test function from Microsoft EXCEL^TM^ (2016) was used for performing statistical analysis (α = 0.05). The deliverer also performed data analysis, and therefore the results were not masked during this study. However, the person timing the experiment was independent, and did not make any observations. Also, to prevent potential biases associated with using different observers, only one observer was used for conducting all the tests.

## Results and discussion

**Fig 3:**
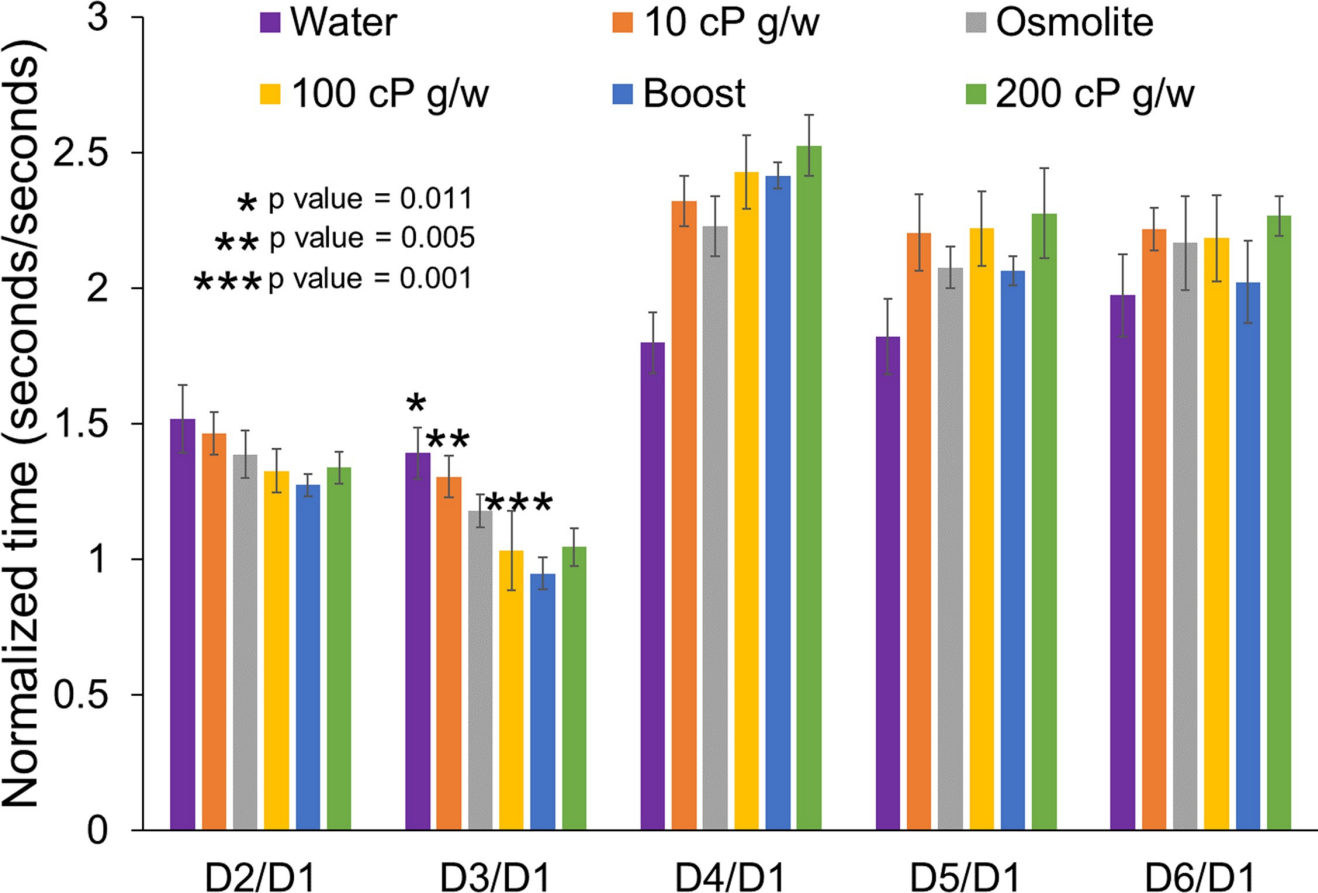
Normalized time required by each surrogate device design. Time required by device normalized with the time required by D1 for six different fluids. The data are then grouped together for different diets with the same surrogate device.

Fig 3 shows the dispensing time for the different devices, normalized by the dispensing time for the surrogate device D1. The normalized time for D2 is greater than 1 for all diets, owing to a higher flow resistance introduced by the ISO 80369–3 connector's smaller inlet diameter section. The lower normalized dispensing times for surrogate D3 relative to those for D2 (p-values for water, 10 cP glycerin-water, 100 cP glycerin-water shown in Fig 3, for Osmolite, Boost and 200 cP glycerin-water, p-values <<< 0.005) indicate that the influence of the smaller inlet diameter on flow rate can be partially compensated for by a larger diameter in the distal section for thin diets such as water, and can be completely recovered for thicker dietary supplements such as Boost™. The longer normalized dispensing times for all the diets with D4 relative to D2 (p-values for all diets <<< 0.005) are due to the longer (50mm) transition section (Fig 2A and 2B). This increase in time can be partially reduced with an increase in distal diameter (D5). However, a further increase in distal diameter (D6) does not improve dispensing time (p-value = 0.018 for water, p-values > 0.1 for all other diets). This is likely because flow separation (and a subsequent energy loss) in the rapid expansion from the 2.9 mm section to the 6.0 mm section offsets the increase in diameter.

The normalized times in Fig 3 can also be used to understand the effect of increasing fluid viscosity. Changes in device geometry manifested as changes in dispensing time more strongly for the thicker diets than for water. For example, in changing from D3 to D4, which involved lengthening the small-diameter inlet section, the normalized dispensing time for water increased by about 30%, while the normalized dispensing time for the Boost™ diet (100 cP fluid) approximately doubled. The smaller change for the low viscosity water may be caused by turbulence (notably, Reynolds number exceeded 10,000). For turbulent flow, friction along the pipe wall represents a more significant energy loss relative to the geometric variations (e.g. expansions) in the device, making alterations to the device geometry and concomitant reduction in flow rate less pronounced [7]. However, for the more viscous diets the flow is laminar and thus the effect of the geometric variations causing reduction in flow rate becomes more pronounced. Overall, our findings indicate that the ISO 80369–3 connector with its short length does not have a significant impact on reducing the flow. Rather, the transition section is the primary contributor impacting the flow rate. Thus, keeping the transition section as short as possible may prevent a reduction in flow in the new ISO 80369–3 connector based G-tube designs.

Table 1 addresses the applicability of Newtonian fluids as surrogates for practical diets, which are typically shear-thinning. To determine whether the surrogates could be considered equivalent to the actual diets, the 95% confidence interval was computed for the difference between the mean normalized dispensing time for the diet and the mean normalized dispensing time for the surrogate. The bounds of the confidence interval were compared with a measure of the smallest effect of practical interest, which we took to be 20% of the mean dispensing time. That is, only increases greater than 20% of the mean dispensing time would be of practical importance. If the confidence interval resided between the +/- 20% bounds of practicality, the surrogate was taken to be equivalent to the diet. The confidence interval limits are shown in Table 1, along with the 20% practicality bounds. It was found that for D3/D1 in the Boost vs. 100 cP g-w comparison, the upper bound of the confidence interval extended slightly beyond the 20% limit. Otherwise, the equivalence criterion was met for both surrogates, for all devices. We conclude that the glycerin-water mixtures constitute useful surrogates for the real diets, for the purpose of evaluating design changes in G-tubes.

**Table 1:** Comparison of Osmolite with 10 cP g-w and Boost with 100 cP g-w.

		Diet		Surrogate		CI of (Diet—Surrogate)		± 20% of Diet Mean	
		Avg. (s/s)	Stdev. (s/s)	Avg. (s/s)	Stdev (s/s)	Lower Bound	Upper Bound	Lower Bound	Upper Bound
Osmolite^TM^ vs 10 cP g-w	D2/D1	1.39	0.09	1.46	0.08	0.00	0.16	-0.28	0.28
	D3/D1	1.18	0.06	1.30	0.08	0.06	0.20	-0.24	0.24
	D4/D1	2.23	0.11	2.32	0.09	0.00	0.19	-0.45	0.45
	D5/D1	2.08	0.08	2.20	0.14	0.01	0.25	-0.42	0.42
	D6/D1	2.17	0.17	2.22	0.08	-0.07	0.17	-0.43	0.43
Boost^TM^ vs 100 cP g-w	D2/D1	1.27	0.04	1.33	0.08	-0.02	0.12	-0.25	0.25
	D3/D1	0.95	0.06	1.03	0.15	-0.04	0.21	-0.19	0.19
	D4/D1	2.42	0.05	2.43	0.13	-0.10	0.12	-0.48	0.48
	D5/D1	2.06	0.05	2.22	0.14	0.04	0.27	-0.41	0.41
	D6/D1	2.02	0.15	2.18	0.16	0.01	0.32	-0.40	0.40

The study had the limitations—that only the 24 Fr G-tube size was assessed, and the device surrogates were simplistic representations of the actual devices. Minor imperfections in the geometries resulting from the 3D printing process were assumed to have a negligible effect on the study. Batch to batch variability in 3D printing process examined on the same day, and with the same batch of diets was found to be minimal (S1 Table in S1 File). As inter-observer variability was shown to increase for the less viscous diets (e.g. water), a single observer was used for all the experiments reported here. It should also be emphasized that because these devices are not actual medical devices, caution should be exercised while making clinical interpretations from our study.

## Conclusions

Based on our findings, we believe that G-tube flow rate can be optimized by considering the following:

- Fluid suspensions possessing the viscosity of clinically relevant diets should be considered in device flow rate testing because of the higher sensitivity of the more viscous diets to changes in geometry.
- Glycerin-based Newtonian fluids can effectively and reproducibly represent commercial diets.
- The length of small-diameter inlet sections should be made as short as possible, as they can reduce the flow rate.
- Increasing the distal diameter can increase the flow rate and help offset the slowing of flow rates caused by the smaller inlet sections. This enhancement is limited, since increases in distal diameter beyond a certain value can result in actual decreases in flow, due to losses in the rapid expansion occurring within the device.

The above technical considerations and the 3D printed design methodology proposed here may be used to aid G-tube manufacturers in designing their new ISO 80369–3 devices by considering design implications on dispensing times.

## Supporting information

S1 File Supporting information reports batch to batch variability in the 3D printed devices, and results from intra and interobserver variability. (DOCX)
