# Supplementary material for: Technical considerations for medical device manufacturers when designing gastrostomy tubes (G-tubes) using the new ISO 80369-3 connector
Source: PLoS One. 2020 Jul 30;15(7):e0236644. doi: 10.1371/journal.pone.0236644 (PMC7392286; doi:10.1371/journal.pone.0236644)
Supplement: S1 File — (DOCX) [file pone.0236644.s001.docx]

Supporting Information

for

**Technical considerations for medical device manufacturers when designing gastrostomy tubes (G-tubes) using the new ISO 80369-3 connector**

Suvajyoti Guha*^a^, Alexander Herman^a^, Luke Herbertson^a^, Mark J. Antonino^b^, Joshua S. Silverstein^b^, Jeffrey Cooper^b^, Matthew R. Myers^a^

^a^ Office of Science and Engineering Laboratories, Center for Devices and Radiological Health, U.S. Food and Drug Administration

^b^ Office of Product Evaluation and Quality, Center for Devices and Radiological Health, U.S. Food and Drug Administration

*Corresponding Author

Suvajyoti Guha, PhD

Division of Applied Mechanics,

Center for Devices and Radiological Health,

U.S. Food and Drug Administration,

10903 New Hampshire Avenue, Silver Spring, MD 20993

Email: [Suvajyoti.Guha@fda.hhs.gov](mailto:Suvajyoti.Guha@fda.hhs.gov)

**Table S1.** Batch to batch variability in 3D printing process of devices obtained by comparing with the same batch of two diets (water and Osmolite) on the same day.

| Diets |  | Batch 1 | | Batch 2 | | |  |
| --- | --- | --- | --- | --- | --- | --- | --- |
|  |  | Avg (s/s) | Stdev. (s/s) | | Avg (s/s) | Stdev. (s/s) | p values |
| Water | D2/D1 | 1.444 | 0.545 | 1.565 | | 0.134 | 0.199 |
|  | D3/D1 | 1.323 | 0.533 | 1.445 | | 0.135 | 0.080 |
|  | D4/D1 | 1.761 | 0.535 | 1.817 | | 0.151 | 0.734 |
|  | D5/D1 | 1.760 | 0.545 | 1.932 | | 0.136 | 0.072 |
|  | D6/D1 | 2.017 | 0.529 | 2.017 | | 0.529 | **0.005** |
| Osmolite | D2/D1 | 1.297 | 0.427 | 1.445 | | 0.464 | 0.054 |
|  | D3/D1 | 1.100 | 0.400 | 1.217 | | 0.476 | 0.087 |
|  | D4/D1 | 2.145 | 0.538 | 2.299 | | 0.469 | 0.596 |
|  | D5/D1 | 1.999 | 0.414 | 2.121 | | 0.487 | 0.877 |
|  | D6/D1 | 2.131 | 0.703 | 2.057 | | 0.162 | 0.086 |

**Table S2.** Interobserver variability for a limited number of geometries (fastest and slowest) and diets (thinnest and thickest) performed with a specific batch of 3D printed devices, and diets, demonstrates that for diets that dispense rapidly there can be statistical difference in the results. Therefore, the same individual observed for all data set presented in the article. The inter-observer variability (measured by differences in dispensing time for different observers) exceeded the intra-observer variability (measured in terms of standard deviation) in most cases.

|  |  | Observer 1 | | Observer 2 | |  |
| --- | --- | --- | --- | --- | --- | --- |
| Devices | Diets | Avg. (s) | Stdev. (s) | Avg. (s) | Stdev. (s) | p values |
| D1 | Water | 2.217 | 0.015 | 2.013 | 0.101 | **0.026** |
|  | Boost | 22.553 | 0.231 | 22.767 | 0.408 | 0.474 |
| D4 | Water | 4.027 | 0.087 | 3.777 | 0.076 | **0.020** |
|  | Boost | 54.777 | 0.090 | 54.820 | 3.787 | 0.985 |
